# Clinical factors associated with severe hypophosphataemia after kidney transplant

**DOI:** 10.1186/s12882-021-02624-3

**Published:** 2021-12-09

**Authors:** Maximilian R. Ralston, Karen S. Stevenson, Patrick B. Mark, Colin C. Geddes

**Affiliations:** 1 Glasgow Renal & Transplant Unit, Queen Elizabeth University Hospital, 1345 Govan Road, Glasgow, G51 4TF UK; 2 Institute of Cardiovascular & Medical Sciences, University of Glasgow, Glasgow, UK

**Keywords:** Kidney transplantation, Hypophosphataemia, Hyperparathyroidism, Graft function

## Abstract

**Background:**

The mechanism by which hypophosphataemia develops following kidney transplantation remains debated, and limited research is available regarding risk factors. This study aimed to assess the association between recipient and donor variables, and the severity of post-transplantation hypophosphataemia.

**Methods:**

We performed a single-centre retrospective observational study. We assessed the association between demographic, clinical and biochemical variables and the development of hypophosphataemia. We used linear regression analysis to assess association between these variables and phosphate nadir.

**Results:**

87.6% of patients developed hypophosphataemia. Patients developing hypophosphataemia were younger, had a shorter time on renal replacement therapy, were less likely to have had a parathyroidectomy or to experience delayed graft function, were more likely to have received a living donor transplant, from a younger donor. They had higher pre-transplantation calcium levels, and lower alkaline phosphatase levels.

Receipt of a living donor transplant, lower donor age, not having had a parathyroidectomy, receiving a transplant during the era of tacrolimus-based immunosuppression, not having delayed graft function, higher pre-transplantation calcium, and higher pre-transplantation phosphate were associated with lower phosphate nadir by multiple linear regression.

**Conclusions:**

This analysis demonstrates an association between variables relating to better graft function and hypophosphataemia. The links with biochemical measures of mineral-bone disease remain less clear.

## Introduction

Hypophosphataemia is a common occurrence following kidney transplantation, occurring in up to 93% of patients, but its mechanism remains debated [1–6]. It was previously proposed that persistent post-transplant hyperparathyroidism, resulting from chronic kidney disease-associated mineral bone disorder (CKD-MBD), led to reduced phosphate reabsorption in the proximal tubule by downregulation of sodium-phosphate cotransporters, resulting in hyperphosphaturia and hypophosphataemia [7, 8]. Further studies have, however, demonstrated a parathyroid hormone-independent mechanism of hypophosphataemia in the post-transplant population [9, 10]. There is an important role played by ‘phosphatonins’, most notably fibroblast growth factor-23 (FGF-23). FGF-23 rises early in CKD-MBD, and also acts by downregulation of sodium-phosphate cotransporters [11–16]. This adaptive response in CKD-MBD to control serum phosphate levels may continue post-transplantation, and promote the development of hypophosphataemia [7, 17–23]. In addition, immunosuppressive drug regimens, in particular glucocorticoids, may also contribute to hypophosphataemia [2, 24–26].

Severe hypophosphataemia may have deleterious effects on musculoskeletal, neurological, haematological, cardiovascular, and respiratory function [3, 6, 27–29]. However, it is unclear to what extent these potential consequences impact patients in the post-transplantation period. Serum phosphate constitutes a small proportion of total body phosphate, and phosphate can be rapidly mobilised from skeletal stores in response to low serum levels, mediated predominantly by serum calcitriol [30]. As a result, significant total body hypophosphataemia can exist with normal or only mildly reduced serum levels.

Despite the common occurrence of post-transplant hypophosphataemia, there are very limited data in the published literature on risk factors for its development. Were it possible to predict the development of severe hypophosphataemia with reasonable accuracy, it may be possible to treat more rapidly, and thus reduce the risk of developing complications in the post-transplant period.

The aim of this study was to investigate which pre- and peri-transplant factors were associated with the risk of developing early post-transplant hypophosphataemia.

## Patients and methods

### Study design & population

We conducted a single-centre retrospective observational study, using prospectively collected data. All adult patients receiving a kidney transplant between 01/01/1999 and 31/12/2018 followed for at least 90 days after transplant were eligible for inclusion. We retrospectively analysed anonymised data from the West of Scotland Electronic Renal Patient Record: recipient sex; age at transplantation; duration of renal replacement therapy prior to transplantation; renal replacement therapy modality at time of transplantation; pre-transplantation serum phosphate; pre-transplantation serum calcium (adjusted for serum albumin); pre-transplantation serum alkaline phosphatase; pre-transplantation serum parathyroid hormone (PTH); having undergone a parathyroidectomy pre-transplantation; in receipt of a prescription for alfacalcidol, calcitriol or cinacalcet at time of transplantation; transplant donor type (deceased vs. living); donor sex; donor age; transplantation date before or after change of standard immunosuppression (see ‘Immunosuppression’ below); presence of delayed graft function. Delayed graft function was defined as the requirement for renal replacement therapy within the first 7 days after transplantation.

Prescription of alfacalcidol, calcitriol, cinacalcet and phosphate supplementation were all at the discretion of the responsible nephrologist, and were not subject to a treatment protocol. General practice would be to prescribe phosphate supplementation if a patient with hypophosphataemia became symptomatic, and so would occur after the phosphate nadir had been reached and therefore not influence the results of our analysis.

### Biochemistry

Serum phosphate was measured at each follow up visit along with serum creatinine. Biochemistry analyses were performed in hospital biochemistry laboratories as a part of routine clinical care. Pre-transplant biochemistry values (serum creatinine, calcium (adjusted for serum albumin), phosphate, alkaline phosphatase, PTH) were calculated as the mean value of the first measurement taken in each of the three months preceding transplantation. Post-transplant serum phosphate results were taken as the first measurement in each of the following time periods after transplantation: 7–10 days; 14–17 days; 21–25 days; 28–33 days; 89–120 days; 179–240 days; 269–330 days; 364–424 days. Hypophosphataemia was defined as <0.70 mmol/L. Post-transplant serum creatinine results were taken as the first measurement between 7–10 days after transplantation. The assay platform used by the hospital biochemistry labs to measure PTH changed on 19/07/2010 from Diasorin Liaison, with a normal range of 0.8–5.0 pmol/L, to Abbot Architect, with a normal range of 1.6–7.5 pmol/L. To allow the time periods before and after this change to be included, PTH results are displayed as multiples of the upper limit of normal.

### Immunosuppression

Before 01/01/2007 maintenance immunosuppression consisted of prednisolone, azathioprine and cyclosporine, with recipients perceived to be high immunological risk having induction therapy with monoclonal IL-2 receptor antagonist and maintenance with mycophenolate mofetil rather than azathioprine. After 01/01/2007 standard immunosuppression consisted of monoclonal IL-2 receptor antagonist induction and maintenance with prednisolone, mycophenolate mofetil and tacrolimus [31]. Individual-level data on prescribed immunosuppressive medications was not readily available. This variable was chosen for a number of reasons. Tacrolimus has been demonstrated to reduce renal phosphate wasting in transplant recipients in comparison to a regimen consisting of cyclosporin and azathioprine [24]. Steroid therapy has been shown to contribute to hypophosphataemia after renal transplantation, and steroid dosing differed significantly between the low-dose tacrolimus group and the cyclosporin groups in the ELITE-Symphony study [25, 31]. We hypothesised that these factors could contribute to the prevalence and severity of post-transplantation hypophosphataemia.

### Statistical analysis

We undertook statistical comparison between patients who developed severe hypophosphataemia (≤0.30 mmol/L), those who developed mild-moderate hypophosphataemia (0.31–0.69 mmol/L), and those who did not develop post-transplantation hypophosphataemia. All continuous variables failed a Shapiro-Wilk test of normality, and thus results are presented as median and interquartile range (IQR). We undertook between group comparisons using Kruskal-Wallis testing. *P*-values were corrected for multiple testing using the Benjamini & Hochberg procedure [32].

We then carried out multiple linear regression analysis of the correlation between the independent variables and the post-transplantation phosphate nadir. In order to increase clinical relevance, data on age was analysed per 10 years (rather than per year), and serum alkaline phosphatase levels were analysed per 10 International Units per litre. The data met the assumption of linearity, but demonstrated heteroscedasticity and the residuals were not approximately normally distributed. As a result, we log-transformed the dependent variable (lowest measured serum phosphate level in the first 90 days following transplantation) which provided data which met the above assumptions of the linear regression model. The resulting coefficient estimates were then exponentiated to allow interpretation. A relative importance analysis was then carried out using the metric described in Lindemann, Merenda & Gold [33].

We carried out a post-hoc sensitivity analysis of both the between groups comparison and the multiple linear regression, including only patients receiving their first transplant.

We performed statistical analysis using *R*, running *R* version 4.0.2, using packages *tidyverse, ggplot2, ggpubr, broom, janitor*, *caret* and *relaimpo*, available at http://www.R-project.org.

## Results

Recipients of 1920 kidney transplants were identified. 20 did not have any serum phosphate measurements recorded after transplantation, and so were excluded. A further 62 were excluded due to early graft loss (defined as a return to renal replacement therapy within 30 days of transplantation). Recipients of a total of 1838 kidney transplants were included in the analyses. Of these, 1751 patients had only one transplant in this cohort, 86 had two and 1 patient had three transplants in this cohort.

39.9% of transplant recipients were female, and the median recipient age at transplantation was 48 years. 73.8% of transplants were from deceased donors, 49.5% from female donors, and median donor age was 50 (IQR 39–59) years at donation. Recipients had been on RRT for a median of 1.93 (IQR 0.75–3.79) years prior to transplantation. Median pre-transplant biochemistry results can be seen in Table 1. At the time of transplant 55.2% of patients were prescribed alfacalcidol or calcitriol, 11.2% were prescribed cinacalcet, and 9.7% of patients had undergone a parathyroidectomy pre-transplantation. 30.4% of transplants occurred before 01/01/2007, when standard immunosuppression in the centre changed to a tacrolimus-based regimen as described above. 20.1% of patients had delayed graft function. 10.5% of patients received a prescription for phosphate supplementation in the first 6 months post-transplantation.

**Table 1:** Characteristics of the study population

Variable	All patients (n = 1838)	No. of missing values
Epidemiological		
**Recipient sex (% Female)**	39.9%	0
**Median age at transplant (years [IQR])**	48 (37–57)	0
**Donor sex (% Female)**	49.5%	260
**Median donor age (years [IQR])**	50 (39–59)	282
**Transplant type (% deceased donor)**	73.8%	0
Biochemical		
**Median pre-transplant phosphate (mmol/L [IQR])**	1.64 (1.37–1.96)	49
**Median pre-transplant adjusted calcium (mmol/L [IQR])**	2.40 (2.29–2.50)	172
**Median pre-transplant alkaline phosphatase (U/L [IQR])**	108 (75–173)	56
**Median pre-transplant PTH (xULN [IQR])**	5.8 (3.0–10.1)	53
**Median serum creatinine 7–10 days post-transplant (μmol/L [IQR])**	212 (123–504)	14
Clinical		
**Median duration of RRT pre-transplant (years [IQR])**	1.93 (0.75–3.79)	0
**Modality of RRT prior to transplantation**		8
Haemodialysis	58.9%	
Peritoneal dialysis	28.4%	
None (pre-emptive transplantation)	12.2%	
**Parathyroidectomy pre-transplant (%)**	9.7%	0
**On alfacalcidol or calcitriol pre-transplant (%)**	55.2%	0
**On cinacalcet pre-transplant (%)**	11.2%	0
**Era of immunosuppression (% pre-01/01/2007)**	30.4%	0
**Delayed graft function**	20.1%	0

One thousand six hundred ten patients (87.6%) developed hypophosphataemia (serum phosphate <0.70 mmol/L) within the first 90 days following kidney transplantation, of whom 199 patients (10.8%) developed severe hypophosphataemia (serum phosphate ≤0.30 mmol/L).

### Characteristics of patients developing post-transplantation hypophosphataemia

There were statistically significant differences between the groups of patients who experienced severe, mild/moderate, or no post-transplantation hypophosphataemia in median age at transplantation (45 [35–54] vs. 48 [37–57] vs. 49 [41–59] years, *p* = 0.004), median duration of renal replacement therapy prior to transplantation (1.64 [0.70–2.95] vs. 1.90 [0.75–3.75] vs. 2.55 [0.91–4.43] years, *p* = 0.02), prevalence of pre-transplantation parathyroidectomy (2.0% vs. 8.6% vs. 22.8%, *p* < 0.001), type of graft donation (61.8% vs. 72.8% vs. 89.9% for deceased donation, *p* < 0.001), median age of transplant donor (47 [36–58] vs. 50 [38–58] vs. 56 [45–64] years, *p* < 0.001), and in the incidence of delayed graft function (10.1% vs. 18.0% vs. 42.1%, *p* < 0.001). There were also statistically significant differences between the groups on median pre-transplantation serum calcium (2.42 [2.33–2.51] vs. 2.40 [2.29–2.50] vs. 2.36 [2.22–2.48] mmol/L, *p* = 0.001) and median pre-transplantation serum alkaline phosphatase (86 [64–145] vs. 109 [76–176] vs. 126 [81–187] U/L, *p* < 0.001) (Table 2).

**Table 2:** Characteristics of the study population by severity of hypophosphataemia

Variable	Severe hypophosphataemia (≤0.3 mmol/L, n = 199)	Mild-moderate hypophosphataemia (0.31–0.69 mmol/L, n = 1411)	No hypophosphataemia (≥0.7 mmol/L, n = 228)	***P***-value
Epidemiological				
**Recipient sex (% Female)**	40.2%	40.3%	37.2%	0.69
**Median age at transplant (years [IQR])**	45 (35–54)	48 (37–57)	49 (41–59)	0.004
**Donor sex (% Female)**	46.3%	49.9%	49.5%	0.70
**Median donor age (years [IQR])**	47 (36–58)	50 (38–58)	56 (45–64)	<0.001
**Transplant type (% deceased donor)**	61.8%	72.8%	89.9%	<0.001
Biochemical				
**Median pre-transplant phosphate (mmol/L [IQR])**	1.69 (1.45–1.95)	1.65 (1.38–1.97)	1.56 (1.26–1.94)	0.059
**Median pre-transplant adjusted calcium (mmol/L [IQR])**	2.42 (2.33–2.51)	2.40 (2.29–2.50)	2.36 (2.22–2.48)	0.001
**Median pre-transplant alkaline phosphatase (U/L [IQR])**	86 (64–145)	109 (76–176)	126 (81–187)	<0.001
**Median pre-transplant PTH (xULN [IQR])**	5.7 (3.1–9.5)	5.9 (3.1–10.1)	5.6 (2.3–10.6)	0.53
**Median serum creatinine 7–10 days post-transplant (μmol/L [IQR])**	123 (90–232)	201 (123–467)	522 (302–730)	<0.001
Clinical				
**Median duration of RRT pre-transplant (years [IQR])**	1.64 (0.70–2.95)	1.90 (0.75–3.745)	2.55 (0.91–4.43)	0.016
**Parathyroidectomy pre-transplant (%)**	2.0%	8.6%	22.8%	<0.001
**On alfacalcidol or calcitriol pre-transplant (%)**	57.8%	54.2%	58.8%	0.50
**On cinacalcet pre-transplant (%)**	10.6%	11.7%	7.9%	0.50
**Era of immunosuppression (% pre-01/01/2007)**	24.1%	30.4%	35.5%	0.059
**Delayed graft function**	10.1%	18.0%	42.1%	<0.001

There were no statistically significant differences between the groups on recipient sex, donor sex, whether their transplant was undertaken in the era before or after the switch to a tacrolimus-based standard immunosuppression regimen, or on the prevalence of prescription for alfacalcidol or calcitriol, or of cinacalcet. With regards to biochemical measures, there were no differences between the groups on average pre-transplantation serum phosphate or pre-transplantation serum parathyroid hormone (Table 2).

### Association of variables with post-transplantation phosphate nadir

**Fig. 1:**
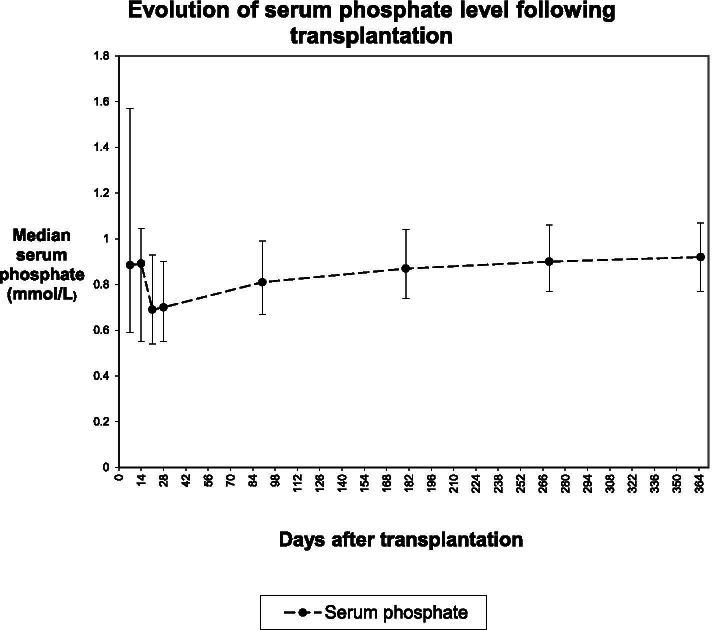
Evolution of serum phosphate level following transplantation

The median serum phosphate nadir in the study population was 0.46 mmol/L (IQR 0.37–0.59), with a range of 0.17–1.66 mmol/L. The phosphate nadir occurred a median of 22 days after transplantation (IQR 10–40). The evolution of phosphate levels in the first year following transplantation is shown in Fig. 1.

By multiple linear regression analysis, receiving a live donor transplant, lower donor age, transplantation following the change to a tacrolimus-based immunosuppression regimen, higher average pre-transplant calcium, higher average pre-transplant phosphate, not having had a parathyroidectomy pre-transplant and not experiencing delayed graft function were all significantly associated with lower serum phosphate nadir following transplantation (Table 3). However, it should be noted that the *adjusted R*^*2*^ = 0.13, suggesting that only 13% of the variance of post-transplantation phosphate nadir was explained by the variables included in this model.

**Table 3:** Results of multiple linear regression model for associations with post-transplantation phosphate nadir

Variable	Exponentiated regression coefficient estimate	***P***-value	Relative Importance
Epidemiological			
**Recipient sex (male)**	1.01	0.601	0.19%
**Age at transplantation (per 10 years)**	1.00	0.864	1.66%
**Donor sex (male)**	1.00	0.883	0.08%
**Donor age (per 10 years)**	1.03	<0.001	9.6%
**Transplant type (live)**	0.89	<0.001	14.66%
Biochemical			
**Mean pre-transplantation phosphate (mmol/L)**	0.94	0.001	4.79%
**Mean pre-transplantation adjusted calcium (mmol/L)**	0.75	<0.001	10.77%
**Mean pre-transplantation alkaline phosphatase (per 10 U/L)**	1.00	0.127	3.34%
**Mean pre-transplantation PTH (per multiple of upper limit of normal)**	1.00	0.243	0.47%
Clinical			
**Duration of RRT pre-transplantation (years)**	1.00	0.498	1.02%
**Parathyroidectomy pre-transplantation**	1.21	<0.001	22.48%
**On alfacalcidol or calcitriol pre-transplantation**	0.97	0.077	0.62%
**Era of immunosuppression (post-01/01/2007)**	0.95	0.052	4.92%
**Delayed graft function**	1.16	<0.001	25.41%

### Sensitivity analyses

A post-hoc sensitivity analysis was carried out, excluding data for second and third transplants. This showed no significant difference in any variable in either analysis.

## Discussion

This is the largest study to date analysing factors predicting hypophosphataemia in the early post-transplant period. We found that factors associated with good early transplant function (living kidney donor, lower donor age, no delayed graft function) and pre-transplant hyperparathyroidism (absence of previous parathyroidectomy and increasing pre-transplant serum calcium and phosphate) were independently associated with lower post-transplant serum phosphate nadir. These findings are important because it may help clinicians anticipate severe hypophosphataemia in patients with these features at the time of transplant. However, our data indicate that the relationships between pre-transplant factors and the development of post-transplant hypophosphataemia are complex and likely to involve factors that we did not measure, given the linear regression *adjusted R*^*2*^ = 0.13. We were surprised that pre-transplant PTH levels were not predictive of post-transplant hypophosphataemia, but this may relate to the fact that patients were on treatments to maintain PTH levels within recommended ranges pre-transplant. It is also now known that FGF-23 is important in maintaining serum phosphate concentration, but since measurement is not part of routine care, it is not included in our study.

Incidence of post-transplant hypophosphataemia was high in this patient cohort (87.6%), in keeping with results from previous smaller studies [7, 17, 28, 34–38]. Lowest phosphate measurements were seen on average 3 weeks after transplantation, and median serum phosphate for the study population then rose gradually throughout the follow-up period to 12 months post-transplantation. This is in keeping with changes over time seen in previous studies [7, 20, 37].

In our study, receipt of a live donor graft, and receipt of a graft from a younger donor were associated with increased incidence and severity of hypophosphataemia. Increasing donor age is known to be a risk factor for poorer allograft function [39–41], as is receipt of a graft from a deceased donor [42]. This may suggest that factors associated with improved graft function put patients at an increased risk of developing post-transplantation hypophosphataemia. This would be consistent with a pathophysiological mechanism of hyperphosphaturia resulting from the metabolic derangement of CKD-MBD, in which better graft function would be associated with increased phosphaturia due to an increased number of functioning nephrons [4, 5]. This may be the explanation for the known association between high serum phosphate levels after transplantation, and increased rate of graft failure [36, 43–48]. This is reinforced by the association between increased incidence of delayed graft function and higher post-transplantation serum phosphate levels seen in our study. These risk factors are similar to those identified in a recently published study [38].

The relationship between hypophosphataemia and biochemical measures of CKD-MBD currently available in clinical practice are less clear from this study. Pre-transplantation serum PTH was not associated with either the absolute development of hypophosphataemia, nor with the post-transplantation phosphate nadir. This is consistent with recent literature that PTH is not the main determinant of post-transplantation hypophosphataemia, and that FGF-23 plays a more significant role in this [4, 17, 19, 20, 22, 23, 49–52]. However, there is some suggestion that persistent hypophosphataemia beyond 1 year post-transplantation may be driven by hyperparathyroidism, as FGF-23 levels have usually fallen significantly before this point [4, 18, 22, 53]. Thus it may be that the follow-up period in this study was too short to capture the impact of persistent hyperparathyroidism on serum phosphate levels.

Interestingly, patients who had undergone a parathyroidectomy prior to kidney transplantation were less likely to develop hypophosphataemia following transplantation, and having had a parathyroidectomy was associated with a significantly higher phosphate nadir post-transplantation. FGF-23 is known to act on the parathyroid glands, predominantly to reduce the expression of PTH [54]. However, the concurrent rise in both FGF-23 and PTH in CKD suggests that the parathyroid glands become unresponsive to FGF-23, and this may be related to uraemia [54, 55]. With the resolution of uraemia following successful kidney transplantation, it may be that the parathyroid glands become FGF-23 responsive again, leading to increased phosphate excretion and lower serum phosphate levels. Those who have undergone a parathyroidectomy prior to kidney transplantation will not experience this change in responsiveness to FGF-23 (owing to the lack of parathyroid glands to act upon), and thus may have a smaller change in serum phosphate levels after transplantation. An alternative explanation would hold that having required a parathyroidectomy may imply a more prolonged course of chronic kidney disease with poorer biochemical control. This in turn may be associated with the receipt of more marginal donor kidneys, and be therefore associated with poorer graft function and higher post-transplantation serum phosphate nadir. FGF-23 is released from osteocytces in bone [56]. Patients with prior parathyroidectomy are more likely to have adynamic bone disease [57]. It appears unknown if adynamic bone disease is subsequently associated with reduced FGF-23 expression, which would be another potential mechanism by which patients with prior parathyroidectomy are at lower risk of hypophosphataemia.

Pre-transplantation serum phosphate levels showed a trend towards a significant association with hypophosphataemia on univariable analysis, but did not meet the pre-specified threshold for statistical significance after correction for multiple testing. However, on regression analysis, increased pre-transplantation serum phosphate levels were associated with decreased post-transplantation phosphate nadir. We feel it is likely that this is a type II error in the univariate analysis, resulting from the relatively smaller severe hypophosphataemia and no hypophosphataemia groups, and the adjustment for multiple testing.

Despite having comprehensive data on a large number of subjects our study has several limitations that should be acknowledged. Firstly, this was a retrospective study from a single centre. However, the use of a comprehensive electronic patient record throughout the period of study meant that few data were missing, and the clinical practice in our unit is consistent with most other transplant centres. Secondly, we did not include measurements of FGF-23 in this study, which has recently been shown to relate to post-transplant hypophosphataemia. However, this is not measured routinely in our clinical practice, and thus could not be included in this retrospective study. Thirdly, although CKD-MBD is a chronic condition, the relevant biochemical parameters were measured in a short period prior to transplantation. However, our method of presenting values as the mean of the first result in each of the preceding 3 months represents a significant improvement on providing a single measurement at time of transplantation. Finally, we have analysed the use of alfacalcidol or calcitriol, and cinacalcet as dichotomous categorical variables, rather than including differences in dose. While including dose may have added a degree of additional nuance, these doses are altered over time, and this would have added significant complexity to the statistical analysis.

Despite these limitations the findings of this study create a stimulus to further research to explore the aetiology and consequences of severe hypophosphataemia post-transplant. It is unclear to what extent post-transplant hypophosphataemia is associated with adverse outcomes, and further research in this area is required. This research should focus on the evaluation of patient-centred outcomes, such as hospitalisations, morbidity (e.g. fractures, graft loss) and mortality. An assessment of the costs of post-transplant hypophosphataemia would also be valuable, including aspects such as prescriptions and hospital admissions, to assess the impact this has on the healthcare system. Phosphate supplementation may exacerbate hyperparathyroidism, increase FGF-23 levels, and even cause nephrocalcinosis [2], and thus research into the effects of supplementation in asymptomatic mild-to-moderate hypophosphataemia is also required. The limited relationship between widely available biochemical parameters and post-transplantation hypophosphataemia suggests FGF-23 measurement in routine clinical practice would be of interest, in order to improve clinical prediction and allow more prompt intervention. Given the link between graft function and hypophosphataemia, histological data on the extent of perioperative tubular injury would add detail, but this would have to be collected from routine, protocolised biopsies, rather than those undertaken by indication, to avoid selection bias. Finally, we have included data on prescription of medications to treat CKD-MBD at the time of transplant, but analysis of the effect of continuing or discontinuing these medications following transplantation would potentially be of value.

In conclusion, this study suggests that variables relating to better kidney graft function are associated with an increased risk of developing post-transplantation hypophosphataemia. It reinforces the complexity of the relationship between CKD-MBD, currently available biochemical measurements, and post-transplantation hypophosphataemia.

## Data Availability

The dataset generated for this study is not publicly available, but is available from the corresponding author upon reasonable request.

## Abbreviations

CKD-MBD: Chronic Kidney Disease Mineral Bone Disease
FGF-23: Fibroblast Growth Factor 23
IQR: Interquartile range
PTH: Parathyroid hormone
